# Functionalized Carbon Materials for Electronic Devices: A Review

**DOI:** 10.3390/mi10040234

**Published:** 2019-04-03

**Authors:** Urooj Kamran, Young-Jung Heo, Ji Won Lee, Soo-Jin Park

**Affiliations:** Department of Chemistry, Inha University, 100 Inharo, Incheon 22212, Korea; malikurooj9@gmail.com (U.K.); heoyj1211@naver.com (Y.-J.H.); lj529@naver.com (J.W.L.)

**Keywords:** carbon nanotubes, graphene, carbon fibres, functionalization, supercapacitors, sensors, inkjet printer inks, flexible wearable devices, electronics

## Abstract

Carbon-based materials, including graphene, single walled carbon nanotubes (SWCNTs), and multi walled carbon nanotubes (MWCNTs), are very promising materials for developing future-generation electronic devices. Their efficient physical, chemical, and electrical properties, such as high conductivity, efficient thermal and electrochemical stability, and high specific surface area, enable them to fulfill the requirements of modern electronic industries. In this review article, we discuss the synthetic methods of different functionalized carbon materials based on graphene oxide (GO), SWCNTs, MWCNTs, carbon fibers (CFs), and activated carbon (AC). Furthermore, we highlight the recent developments and applications of functionalized carbon materials in energy storage devices (supercapacitors), inkjet printing appliances, self-powered automatic sensing devices (biosensors, gas sensors, pressure sensors), and stretchable/flexible wearable electronic devices.

## 1. Introduction

**Figure 1:**
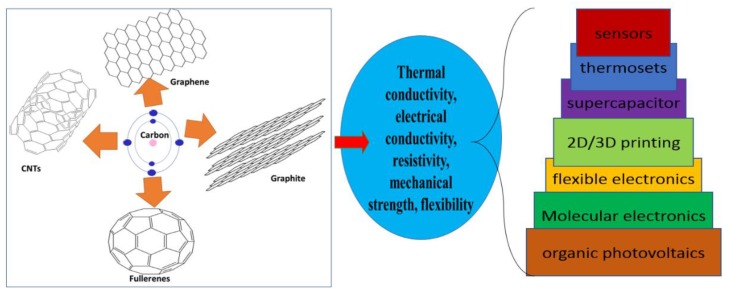
Brief description of various carbon allotropes, their electronic properties and applications in electronic industries.

Carbon is a crucial element, as it is considered the sixth most abundant element in the universe, fourth most ordinary species in the planetary system, as well as the seventeenth most dominant component in the earth’s crust [1]. The overall assessed relative abundance of carbon is 180–270 ppm [2]. The existence of some carbon allotropes, such as graphene, fullerenes (C60), carbon nanotubes (CNTs), amorphous carbon, diamonds (NDs), and londsaleite [3], has attracted the interest of scientists in recent years due to their brilliant properties. These include wide specific surface area, good electrical conductance, high electro-chemical stabilization ability, good thermal conductivity, and high mechanical resistance, which can fulfill the energy-storing needs in electronic-based industries [4,5,6,7,8]. A brief outline of carbon allotropes, their essential physio-chemical properties, and applications in various fields are mentioned in Figure 1.

The progress in electronic industries has reorganized expertise in electro-communications, automation, and computational industries, all of which have a tremendous influence on various aspects of human lives [9]. Previously, the silicon industry was established to develop more effective, compressed, dense, rapid, and stable electronic devices [10]. These advances in silicon technology were synchronized with the simultaneous minimization of electronic devices in order to make them more effective. Despite this progress in silicon-based devices, further advancement is inadequate to satisfy the various scientific and technical aspects for electronic devices because of resource limitations [11]. Recently, researchers have found that carbon-based materials are an alternative to silicon technology and have proven to be very effective to fabricate electronic devices with superior performance.

The importance of carbon-based materials in electrical devices is due to their tremendous properties. Graphene exhibits low weight, efficient electric as well as thermal conductivity, high specific tunable surface area (up to 2675 m^2^∙g^−1^), superior mechanical properties, with a Young’s modulus (YMs) of almost 1 TPa, and high chemical stability [12,13,14]. CNTs exhibit efficient mechanical characteristics, with a YMs in the range of 0.27–1 TPa and high mechanical strength (11–130 GPa) [15,16]. A literature survey indicated that CNTs possessed thermal and electrical conductance up to 3,000 Wm∙K^−1^ [17] and 1800 S∙cm^−1^ [18], respectively. NDs exhibit sp^2^ and sp^3^ carbon layers, which contribute to their high durability [19,20]. These tremendous properties of carbon-based materials result in various applications in an electronic devices, energy production (Li-ion batteries, solar-fuel cells) [21], energy storage devices, and environment-protection-related appliances [22,23,24,25,26,27,28,29,30,31,32,33,34]. Therefore, the utilization of carbon-based materials instead of silicon provides an innovative and new technological platform in the electronic industry. Recently, graphene, CNTs, fullerene derivatives (FDs), carbon nanofibers (CFs), NDs, 2D graphene layers, and carbon foams, have been widely used in electronics; their applications include supercapacitors, thermosets, molecular electronics, organic photovoltaics, gas-sensor, biosensors, 2D or 3D printers, flexible electronic wearable electronics, wireless telecommunication systems, electromagnetic-wave (EM) absorber materials in reverberation chambers, fillers in EM shielding cementitious materials, EM wave propagating tuner materials [35,36,37,38,39,40,41,42,43,44,45,46,47,48,49,50,51,52,53,54]. However, the direct use of carbon-based materials without any modification and functionalization lead to frequent aggregation, and insolublilization sometimes limits their industrial applications. Therefore, from a practical perspective for industrial applications, it is extremely necessary to fabricate functionalized materials with tuneable physio-chemical surface properties in order to improve their performance. For this purpose, many methodologies have been proposed to produce doped and functionalized carbon-based materials for energy and electronic applications [55,56,57].

Herein, we focused on the strategies of functionalization or modification of carbon-based materials and highlighted their applications in electronic devices, including supercapacitors, inks, inkjet/3D printing appliances, biosensors, gas sensors, and flexible electronics wearable.

## 2. Methodology

### 2.1. Functionalized Carbon-Based Electrode Materials

#### 2.1.1. Activation Method

**Figure 2:**
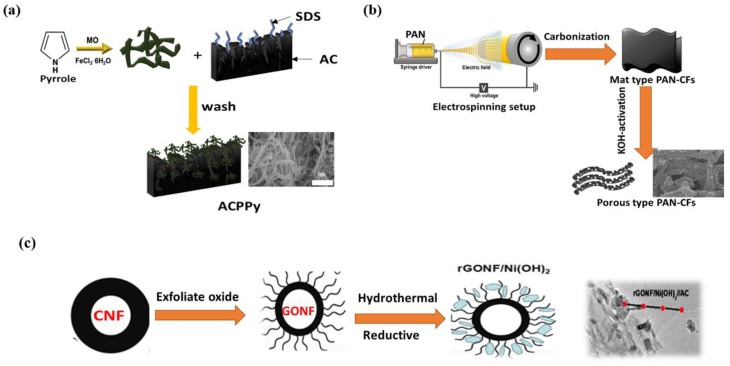
(**a**) Schematic layout of synthesis of activated carbon/polypyrrole (AC-PPy) composites, reproduced with permission from [58], published by Elsevier, 2018; (**b**) synthetic scheme of porous polymer-based carbon fibers (PPCFs) preparation process by electrospinning technique, reproduced with permission from [64], published by Elsevier, 2019; and (**c**) general synthetic layout for rGONF/Ni(OH)_2_ fabrication, reproduced with permission from [78], published by ACS Publications, 2016.

A carbon-based electrode hybrid material is obtained by polymer functionalization, metal oxide (MO) doping, or by activation with activating agents. The polymer-based functionalization of activated carbon (AC) for supercapacitor electrodes has been reported in literature. Lee et al. [58] synthesized tubular polypyrrole-(T-Ppy) functionalized pitch-based AC to obtain a hybrid composite material (AC/Ppy) for a supercapacitor electrode; the synthetic layout is mentioned in Figure 2a. Different contents ratios (1:0.5, 1:1, 1:2, 1:4) of T-Ppy were loaded onto AC [59,60]. The electrode was prepared by dipping a small nickel piece in a slurry of 80% Ppy/AC hybrid composite and 10% conductive material, with 10% (PVDF:NMP) as a binder solvent. Vighnesha et al. [61] fabricated activated AC (the AC was derived from coconut shell) and functionalized by polyaniline. KOH-activated AC was obtained by polymerization of aniline [62]. A hybrid electrode was prepared by mechanical mixing of AC and PANi with various ratios and N-methyl pyrrolidone and PVDF as a binder, followed by loading on a current-collector steel.

#### 2.1.2. Electrospinning Technology 

Polypyrrole (Ppy) and polyacrylonitrile (PAN) have also been used to functionalize CFs. In this regard, Tao et al. [63] fabricated a composite hybrid material for flexible supercapacitors by in-situ growth followed by conductive wrapping. The fabrication steps included the electrodeposition of MnO_2_ nanoparticles on cleaned CFs, which was followed by the wrapping of a conductive Ppy layer. The supercapacitor electrode was prepared by fixing the as-synthesized hybrid material (Ppy-MnO_2_-CFs) to a supercapacitor in a sandwich structure, with PVA/H_3_PO_4_ as the membrane as well as an electrolyte solution between the electrodes. Furthermore, electrospinning technology was used to prepare PAN-functionalized CFs (APCFs) [64], as mentioned in Figure 2b. The pristine PAN-CFs material was chemically activated by KOH at different temperatures (600–1000 °C) under N_2_ atmosphere. The capacitance test was performed in a 1.0 M Na_2_SO_4_ electrolyte and the coated working electrode was prepared in the same way as mentioned in Ref. [58]. 

#### 2.1.3. Greener and Rotational Hydrothermal Method

Recently, Cakici et al. [65] utilized carbon fiber fabric (CFF) to prepare a hybrid composite with MnO_2_, named CFF-MnO_2_. A greener hydrothermal approach was used for the doping of MnO_2_ nanoparticles on CFF. Briefly, the polymer sizing on CFF was first eliminated by annealing at a specified temperature under Ar atmosphere. Fine CFF was then autoclaved with a 6 mM KMnO_4_ solution at 175 °C. Different samples were prepared under same conditions with varying times. The electrochemical test was obtained using a 3-electrode electrochemical cell system, with a 1 M Na_2_SO_4_ electrolyte, Ag/AgCl as a reference electrode, and the prepared fabric material (CFF/MnO_2_) itself as a working electrode.

Graphene-based modified hybrid electrode materials have also been reported. Ke et al. [66] demonstrated different approaches for fabricating a reduced graphene oxide (rGO)-doped Fe_3_O_4_ nanocomposite electrode material based on strong electrostatic interactions between oppositely charged graphene oxide (GO) and Fe_3_O_4_ via a rotational hydrothermal approach with some modifications. A modified Hummers’ process was used to convert graphite into GO. However, Li et al. [67] fabricated a hydrothermally reduced graphene-(HRG) decorated MnO_2_ composite on the basis of previous reports [68,69,70,71,72] via a hydrothermal approach. In this case, GO was prepared by a modified Hummers’ method [73,74]. The influence of polyaniline-functionalized graphene sheets (GS) doped with various MO (RuO_2_, TiO_2_, and Fe_3_O_4_) on the supercapacitance was also studied [75]. The graphite was converted into graphitic oxide by Hummers’ method. Hydrogen thermal exfoliation of graphitic oxide lead to the formation of hydrogen-exfoliated graphene sheet (HEG), which was further functionalized by HNO_3_. Sawangphruk et al. [76] invented a hybrid pseudo-supercapacitor based on double layer graphene functionalized with polyaniline and doped with silver nanoparticles deposited on flexile type CFs paper. Wang et al. [77] successfully fabricated q hybrid material containing Ni(OH)_2_, GS, and GO; GS was obtained by an exfoliation-expansion method and GO was obtained by a modified Hummers’ method. GO/DMF and GS/DMF were identically mixed with a Ni(Ac)_2_ solution for hydrothermal autoclave treatment at 80 °C for 10 h and coated on a pseudo-capacitive electrode. The composite material was dispersed in an ethanol-PTFE solution and hydrated into nickel foam through compression at 80 °C. Electrochemical measurements were conducted on the foam sample. Similarly, Zhang et al. [78] produced GO nanofibers (GONFs) and GO nanoribbons (GONRs) from carbon nanofibers (CNFs) using Hummers’ method. The growth of crystalline Ni(OH)_2_ nanoplates and reduction of prepared GONFs and GONRs were obtained via a hydrothermal approach. Specifically, the electrode composite material was synthesized by mixing N_2_H_4_·H_2_O (35%), NH_3_·H_2_O (28 wt%), and Ni(NO_3_)_2_ (0.4 M) with 2 mg∙mL^–1^ aqueous solution containing either GONF, CNF, or GONR. After sonication, the mixture was hydrothermally annealed under certain conditions. The composites were named rGONF/Ni(OH)_2,_ rGONR/Ni(OH)_2_, and CNF/Ni(OH)_2_. The general schematic layout and morphology of the composites are mentioned in Figure 2c. 

### 2.2. Functionalized Carbon-Based Inks and Inkjet Printer Materials

#### Solvent Exfoliated Method

**Figure 3:**
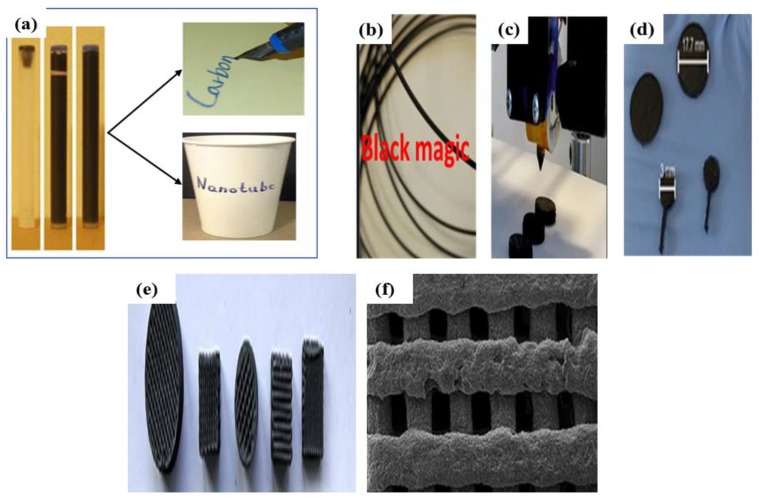
(**a**) Water-based carbon nanotubes ink dispersion in cartridges after 7 days, and direct writing above paper and cup using fountain pen, reproduced with permission from [86], published by Elsevier, 2014; (**b**) 3D printable graphene/PLA, (**c**) 3D printing process, (**d**) various printed 3D electrodes, reproduced with permission from [87], published by Nature, 2017; (**e**) morphology, (**f**) and FESEM images of different 3D graphene-based structure, reproduced with permission from [88], published by Elsevier, 2018.

According to a literature survey, carbon allotropes are used in formation of various inks and hybrid materials within inkjet printing devices [79,80]. For this purpose, Secor et al. [81] formulated ink from graphene by using the solvent exfoliated method [82]. Briefly, graphene was dispersed efficiently in ethanol with a stabilizing polymer, ethyl cellulose (EC). The obtained pure graphene-EC powder was mixed with terpineol to improve the dispersion and obtain smooth printing. The formulated graphene-based ink was utilized for gravure printing by using a flooding doctoring printing system. Similarly, Torrisi et al. [83] formulated a graphene-based printable ink by ultrasonic exfoliation of graphite flakes in an NMP solvent; the obtained mixture was centrifuged to eliminate graphite flakes. A pristine GS-based ink was formulated by Gao et al. [84] through the exfoliation of graphite flakes in a mixture of EC and cyclohexane through ultrasonication under supercritical CO_2_. On the other hand, CNTs are also used to synthesize inks. Homenick et al. [85] fabricated an ink from SWCNTs, which was used in the formation of a SWCNT transistor on a test chip. The scintillation vial was charged by ultrasonication with IsoSol-S100 and toluene. The polymer (PFDD) and SWCNTs were mixed in appropriate ratios and the obtained ink was deposited on a silicon chip through optimized print waveform (20 μm) fitted in an inkjet printer. Multiple layers of the SWCNT-functionalized ink were printed by rinsing the chip several time with toluene within the inkjet printer. In the same way, a SWCNT transistor was prepared on SiO_2_ wafers. The easiest approach for this, using a water-based ink, was reported by Han et al. [86], with a mixture of SWCNTs and sodium dodecyl-benzene sulfonate (SDBS). The SWCNTs and SDBS surfactant with ratio of 1:2 were mixed in 10% distilled water by ultrasonication and probe sonication for 2 h. The fabricated inks were put in a cartridge for a month to obtain a well-dispersed ink. The writing efficiency of the water-based SWCNTs ink was checked on a paper and on a curved cup, as shown in Figure 3a. The primary resistivity of the SWCNTs-ink-coated filter paper was under 20 kV∙cm^−1^.

Further improvements on three-dimensional (3D) printed designs were obtained by Foster et al. [87]. They invented a 3D printed design with the help of a Rep-Rap printer by utilizing a graphene/PLA filament named “black magic”, as shown in Figure 3b; this filament possessed a conductance of 2.13 S∙cm^−1^. The electrochemical test was conducted using a 3-electrode-based system with a printed 3D graphene/PLA anode as the working electrode and calomel and platinum electrodes as the reference electrodes. You et al. [88] fabricated a well-dispersed graphene-based ink for 3D printers. This ink was prepared by ultrasonication of graphene with EGB to achieve a homogenized graphene dispersion, which was further mixed with a solution of DBP and PVB in ethanol medium (2.5:1 ratio), followed by volatilization of ethanol to obtained 3D slurry inks for printing. They also prepared graphene-based filaments, which were used as primary blocks for 3D structures. The different 3D morphological structures with the graphene filaments were prepared via a 3-axis robocasting system and are mentioned in Figure 3e,f. Sarapuk et al. [89] investigated the influence of dispersing agents on the properties of inks. Graphene nanoplates were added into a mixture of glycol and ethanol (1:1) followed by sonication. A functional polymer (AKM-0531) was then added as a dispersing agent to improve the effectiveness of the prepared inks.

### 2.3. Functionalized Carbon Hybrids for Sensors 

#### 2.3.1. Immobilization, Direct and In-Situ Methods

Li et al. [90] fabricated a composite material for biosensor electrodes through a facile method. The GO was produced from graphite and reduced to rGO using NaOH (Hummers’ method) [91]. The rGO/Pd hybrid was obtained by mutual sonication of the rGO suspension and palladium acetate [92]. The immobilization of lacasse was realized through self-polymerization of dopamine, leading to poly-dopamine, and subsequently, the formation of a PDA-Lac-rGO-Pd hybrid composite. The modified biosensor electrode was developed by polishing a glass carbon electrode (GCE) with alumina and coating the GCE with the PDA-Lac-rGO-Pd hybrid material. Hassan et al. [93] also prepared a functionalized graphene-based glucose sensor through the immobilization method. The GO was prepared by a modified Hummers’ method using graphite flakes, while the functionalization and reduction of GO were performed by adding PIL and hydrazine [94]. The obtained GO sheet was coated on a hot Pt wire by dipping [95,96]. The glucose oxidase (GOD) enzyme was electrostatically introduced above the GO-metal wire. The electrochemical test of the prepared GOD-graphene biosensor was conducted with Ag/AgCl as a reference electrode. 

**Figure 4:**
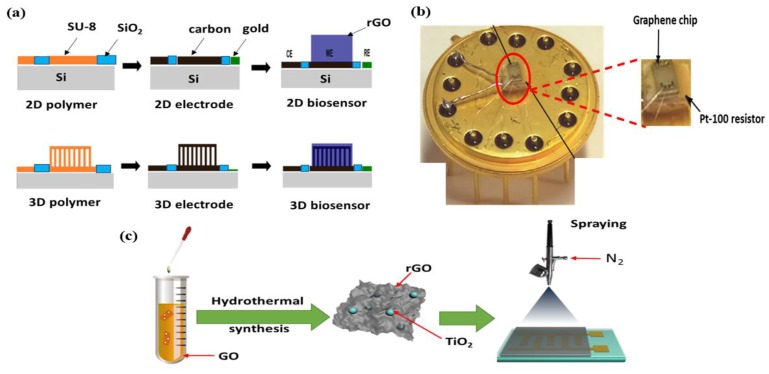
(**a**) Schematic illustration of 2D and 3D carbon-based biosensors, reproduced with permission from [97], published by MPDI, 2018; (**b**) graphene-based sensor chip above holder reproduced with permission from [100], published by Elsevier, 2016; and (**c**) synthetic layout of rGO-TiO_2_ thin-film sensor, reproduced with permission from [105], published by Elsevier, 2017.

Hemanth et al. [97] fabricated a bio-functionalized graphene 3D carbon electrode biosensor. The reduction and functionalization of Hummers-modified GO was obtained by treatment with branched polyethylenimine (PEI) in a single reaction [98]. Further modification and induction of biosensor ability in PEI-GO was carried out by adding ferrocene carboxylic acid; after combing with a biosensing enzyme, the hybrid material was coated on an electrode. A brief schematic layout is shown in Figure 4a, where the biosensor is prepared by coating WE area of 2D and 3D micro-electrodes chip with a solution of the hybrid material (ferrocene-modified RGO-PEI). A similar method was used to prepare a glucose-based biosensing electrode with a GOD (glucose oxidase) enzyme solution. In the case of carbon-based gas sensors, Liu et al. [99] investigated a ZnO-rGO hybrid material for NO_2_ gas sensors. The rGO was prepared from graphite flakes powder by Hummers’ method, and was in-situ decorated with ZnO nanoparticles. The hybrid ZnO-rGO composite material was reduced by hydrazine hydrate. The composite hybrid was mixed with DMF suspension to obtain a DMF-ZnO-rGO hybrid suspension, which was then coated on ceramic substrate electrodes; this was used to test the NO_2_ gas-sensing properties with a static test system.

#### 2.3.2. Thermal Annealing and Hydrothermal Methods

Novikov et al. [100] used graphene films to fabricate a NO_2_-based gas sensor. The graphene film was grown by annealing a 4H-SiC crystal under Ar atmosphere at 1700 °C. Laser photolithography was used to form a bar-shaped design on the graphene surface. The minimum and suitable resistance contacts were obtained via double step metallization, which was carried out by e-beam evaporation and lift-off photolithography [101]. The prepared graphene-based sensor chip was placed in a prototype portable device i.e., above a holder having a platinum resistor as a heater (platinum was chosen due to its minimum thermal inertia). The sample was subjected to rapid thermal cycling, as mentioned in Figure 4b, and the NO_2_ gas adsorption was monitored. Huang and Hu [102] also investigated a rGO-based polyaniline hybrid (rGO-PANI) for the detection of ammonia (NH_3_). A single layer of GO was obtained by ultra-sonication of GO paper in distilled water. The monomer for aniline polymerization (GO-MnO_2_) was prepared by direct reaction with KMnO_4_ and thermal annealing [103,104]. The electrodes for the sensor device were synthesized via a standard microfabrication approach, in which a micro syringe was utilized for the deposition of a rGO-PANI hybrid ethanol solution within the electrode gap, generating a rGO-PANI bridge. Ye and Tai [105] successfully invented a novel rGO-based TiO_2_ thin-film sensor; the brief schematic layout is shown in Figure 4c. The hybrid reduced graphene oxide based TiO_2_ composite (rGO-TiO_2_) was prepared by ultrasonic dispersion of rGO and TiO_2_ with a ratio of 1:96 in a hydrothermally treated mixture of TIP, HCl, and DI water. The prepared rGO-TiO_2_ composite material was deposited on a micro-electrode. This rGO-TiO_2_ thin-film sensor was applied for the detection of NH_3_.

### 2.4. Carbon-Based Materials for Wearables Electronics

#### 2.4.1. Impregination, Thermal Annealing, and Spray Deposition Methods

**Figure 5:**
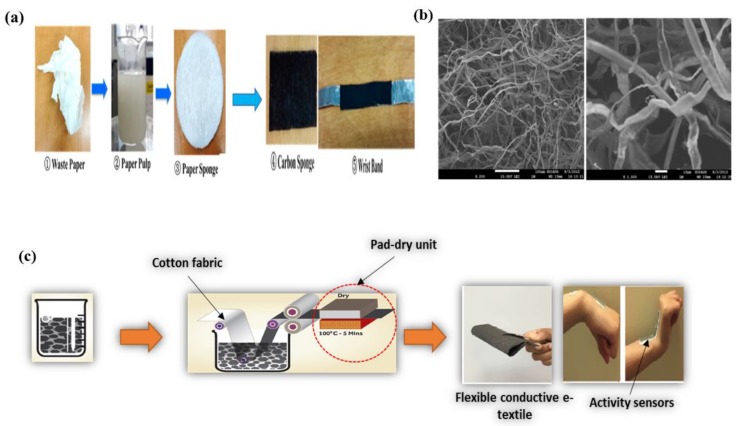
(**a**) Schematic illustration of fabrication of multifunctional wrist band made of CS/PDMS composite, (**b**) SEM images of carbon sponge prepared from paper waste at different magnifications, reproduced with permission from [110], published by ACS Publications, 2016; and (**c**) schematic illustration of scalable formation of reduced graphene oxide-based wearable e-textile, reproduced with permission from [111], published by ACS Publications, 2017.

The applications of carbon allotropes in the field of wearable, flexible, and stretchable electronics are noteworthy. Previously, SWCNT-tissue-paper-based flexible wearable pressure sensor was synthesized by impregnation and thermal annealing [106]. The fabrication procedure involved the mixing of a SWCNT suspension with tissue paper, evaporating the water, and coating the dried tissue paper/SWCNTs solid on a substrate to obtain a resistance of up to 12.6 kΩ. Then, a prepared PDMS layer was poured onto a glass slide and cured by heating. The PDMS layer (300 μm) was deposited on a PI-coated Ti/Au electrode via metal shadow masking. The pressure sensor device was prepared by coating the active SWCNTs/tissue paper on the PDMS/PI surface deposited on an alumina wire; this was connected to an electrode pad with Ag epoxy for electric networking. On the other hand, Wang and Loh [107] synthesized a type of nanocomposite sensing material based on MWCNTs. A thin film was prepared via airbrushing; this technique has been reported in literature [108,109]. Briefly, the dispersion (MWCNTs:distilled water) and Poly(sodium 4-styrenesulfonate):N-Methyl-2-pyrrolidone (PSS:NMP) were mixed to obtain a latex solution. This ink solution was sprayed onto a glass microscopic slide and annealed at a specified temperature to improve its electrical and mechanical characteristics. The annealed thin film was sandwiched among layers of two-sided Fe on an adhesive fabric and pressed by an iron heat presser. The prepared MWCNTs-sensor was cut into small pieces for electrochemical experimental tests. Li et al. [110] invented a multifunctional wristband fabricated from flexible carbon-sponge polydimethylsiloxane composites (CS/PDMS). The brief synthetic outline is clearly mentioned in Figure 5a, the CS conductive material was synthesized from tissue paper waste through ultrasonication in distilled water followed by freeze-drying and pyrolytic heat treatment. A polydimethylsiloxane resin and curing agent were mixed with the prepared CS. After mixing and vacuum drying, the CS was cut into sheets and coated with aluminum foil and silver paste to obtained conductive electrodes, which were then encapsulated by PDMS elastomers. The sensors were finally fixed on wristbands and sports shoes to detect their performance.

#### 2.4.2. In-Situ Chemical Reduction and Full-Solution Methods

Karim et al. [111] fabricated a rGO-based wearable e-textile device. The brief synthetic protocol is presented in Figure 5c. The in-situ chemical reduction method was used to reduce GO; it involved the addition of PSS in a GO suspension followed by stirring. The prepared mixture was transferred to a round-bottom flask that was fixed in an oil bath, then ammonia and Na_2_S_2_O_4_ were added for the reduction of GO. A dispersion of rGO ink was formed with distilled water. The e-textile wearable device was prepared by the pad-drying method, where the textile fabric is dipped/padded several times (1 to 10) in the rGO suspension (pick-up was 80% of textile fabric) in order to improve the electrical conductance of the graphene-based textile fiber. Wu et al. [112] also synthesized a smart e-textile wearable electronic generator based on polyester/Ag nanowires/graphene core-shell nanocomposites via a full-solution process. Plasma-treated textiles were strained with a tension of approximately 20 N. Then, a Ag nanowire solution was pipetted and a blade was used to coat the textile surface via a bottom-up approach. After that, the textile-Ag nanowire was coated with a dispersed GO (prepared by modified Hummers’ method) solution. The prepared e-textile was then treated with hydrazine-hydrated vapor for the reduction of the coated GO. For the tribo-electric generator formation, a PMMA/chloroform suspension was blade-coated onto the textile. Similarly, a PI/e-textile was prepared and annealed under Ar atmosphere for the polymerization of polyamic acid within the layers. The PDMS film was obtained by solidification of the e-textile/PDMS.

## 3. Results and Discussion

### 3.1. Supercapacitors

Supercapacitors play a very important role in applications of energy storage devices, for example, in energy storage chips, implantable electronic devices, wire free electronic-sensors, and many others [113,114,115,116,117]. The different electrode configurations of mini-supercapacitors include sandwich, roll-shaped, as well as inter-digital [118]. The tremendous merits of supercapacitors are its higher power density, lengthy life cycle, and inexpensiveness. For commercial applications of supercapacitor, the electrodes are required to exhibit high ionic sorption ability at solid liquid interfaces and rapid charge transfer. For this purpose, scientists have explored various carbon-based materials (AC, CFs, graphene etc).

Lee et al. [58] prepared an AC/Ppy hybrid composite for a supercapacitor electrode. The prepared AC/Ppy composite had a maximum specific capacitance of 82.3 F∙g^−1^, determined by performing a charge/discharge galvanostatic test. The primary stability loss was observed after 1000 cycles due to the instable nature of Ppy. In comparison, Vighnesha et al. [61] fabricated an AC-PANi composite electrode material; the maximum specific capacitance recorded for this composite at 0.5 m A∙g^−1^ was 99.6 F∙g^−1^ by a charge-/discharge test, which was higher than the previously reported value by Lee et al. [58]. A flexible hybrid electrode composite, Ppy-MnO_2_-CFs, displayed a specific capacitance of 69.3 F∙cm^3^ at 0.1 A∙cm^3^ with an energy density (ED) of 0.00616 Wh∙cm^3^, obtained via cyclic voltammetry and a charge/discharge galvanostatic testing system using a PVA/H_3_PO_4_ electrolyte. It shows electro-chemical stability up-to 1000 cycles [63]. Furthermore, the influence of the specific surface area and pore size on a composite material was studied by Heo et al. [64]. In this work, a CF-based composite activated at 1000 °C had a high specific surface area (1886 m^2^∙g^−1^) and reliable pore size (0.021–1.196 cm^3^∙g^−1^). The APCFs-1000 sample showed a specific capacitance of 103 F∙g^−1^ (1 A∙g^−1^) in a 1 M Na_2_SO_4_ electrolyte with stability up to 3000 cycles. This enhanced supercapacitance occurred due to the high specific surface area, suitability of pore size, and effect of heteroatoms among the enhanced double layers. Further improvements in the specific capacitance properties of an electrode material was reported by Cakici and their co-workers for a CFF/MnO_2_ composite that acted as a flexible electrode in a supercapacitor. Three tests were used to verify the electrochemical performance: cyclic voltammetry, charge/discharge galvanostatic test, and electro-chemical impedance spectroscopy. The cyclic voltammetry analysis showed that the prepared materials had a significantly efficient specific capacitance, with a value of 467 F∙g^−1^ (1 A∙g^−1^). The materials also exhibited high electro-chemical stability after 5000 cycles. Additionally, the as-synthesized device showed a very high energy density (approximately 20 Wh∙kg^−1^) at 0.176 kWh∙kg^−1^. This means that this carbon-based composite hybrid material can be used as a potential electrode for commercial energy-saving devices with supercapacitors [65].

Many researchers have worked on synthesizing graphene-based materials for supercapacitor electrodes. Ke et al. [66] fabricated a Fe_3_O_4_-rGO nanocomposite electrode material that exhibits a specific supercapacitance of 169 F∙g^−1^ at 1 A∙g^−1^ (obtained by cyclic voltammeter and galvanostatic charge/discharge tests). The electrode material showed a capacitance retention of above 88% after 100 cycles. In contrast, Li et al. [67] fabricated GO-decorated MnO_2_ (GO/MnO_2_). The supercapacitor fabricated with the GO/MnO_2_ composite showed a specific capacitance of 211.5 F∙g^−1^ at 2 mV∙s^−1^. The charge/discharge studies showed that 75% capacitance stability was maintained after 1000 cycles by utilizing 1 M Na_2_SO_4_ as an electrolytic solution. Further improvements in hybrid materials with high-specific capacitance has been achieved by developing various MO-doped (RuO_2_, TiO_2_, and Fe_3_O_4_) and polyaniline-functionalized GS. The highest specific capacitance recorded was 375 F∙g^−1^ with 1 M sulphuric acid (as an electrolytic solution) and a sweep-voltage speed range of 10–100 Mv∙S^−1^. Despite the high voltage rate, 85% of the capacitance was maintained, proving that this composite material can be used as an excellent supercapacitor [75]. Sawangphruk et al. [76] invented a hybrid pseudo-supercapacitor (AgNP/PANI-Graphene-CFP) that showed a high specific capacitance in a 1 M Na_2_SO_4_ electrolyte, approximately 828 F∙g^−1^ at 1.5 A∙g^−1^ with a capacitive stability up to 97% after 3000 cycles, obtained via a charge-discharge test.

**Figure 6:**
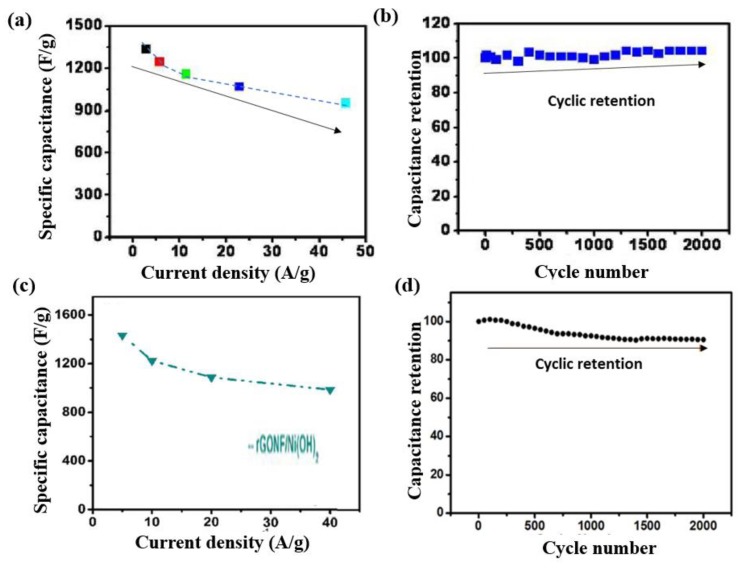
(**a**) Average specific capacitance (SC) at different charge-discharge current densities of 28.6 A∙g^−1^, (**b**) capacitance stability versus cycle number of Ni(OH)_2_/GS, reproduced with permission from [77], published by ACS Publications, 2010; (**c**) SC of rGONF/Ni(OH)_2_ hybrid electrode composites and (**d**) cycling retention of rGONF/Ni(OH)_2_ material, reproduced with permission from [78], published by ACS Publications, 2016.

It is known that exfoliated GO sheets exhibit a wider specific surface area and that they need to be restacked. In order to increase the efficiency of GO sheets, a famous approach is to decorate GO layers using oxide- or hydroxide-based nanomaterials. This technique enables the prepared materials to have enhanced functional applications, for example, increased pseudo-capacitance and also act as a spacer among the GO layers. In this regard, Wang et al. [77] fabricated a hybrid electrode based on Ni(OH)_2_ nanoplates deposited on graphene. The maximum specific capacitance of the Ni(OH)_2_/graphene electrode, as shown in Figure 6a, is approximately 1335 F∙g^−1^ with a 2.8 A∙g^−1^ current density. The capacitance was stable after 2000 charge/discharge cycles at the highest current density tested (28.6 A∙g^−1^), as shown in Figure 6b. A literature survey showed that so far, the most efficient pseudo-capacitive hybrid electrode composite was invented by Zhang et al. [78], named rGONF/Ni(OH)_2_. This composite has the highest recorded specific supercapacitance (1433 F∙g^−1^) at 5 mV∙s^−1^ and maintained approximately 90% of the capacitance after 2000 cycles; this is clearly shown in Figure 6c,d. 

The rGONF/Ni(OH)_2_ hybrid exhibited efficient energy density, and can be utilized at an industrial level for supercapacitor applications. Besides these reported works, as mentioned in Table 1, different modified carbon-based composites have also been studied for supercap, the acitor applications.

### 3.2. Inks and Inkjet Printing Devices

In the previous decades, electronics related to the printing technology have attracted increasing attraction due to the potential of manufacturing inexpensive and large-scale electronic circuits. It is necessary to develop reliable functionalized inks, highly mobile printable semiconductors, conducting inks that consume less energy, and higher-magnification and uniform printing tools, in contrast to conventional techniques [138]. The first work related to the production of conductive inks for inkjet printing based on graphene was conducted by Torrisi and Coleman [139]. They obtained a large amount of GS rapidly by liquid-phase exfoliation in easily printable solvents, including water and other organic liquids. The resulting ink showed stability and easy processability at room temperature and exhibited high batch reproducibility with efficient rheological characteristics for printing. Secor et al. [81] reported on gravure printing of graphene to rapidly form conductive patterns on a stretchable substrate; they fabricated reliable inks and presented printing parameters permitting the synthesis of patterns under magnifications up to 30 μm. The inclusion of a mild heating process resulted in conductive lines with high regularity. These results provide an effective approach for the integration of graphene into large printed areas as well as in flexible electronics. Torrisi et al. [83] investigated a feasible approach for broad-scale synthesis of graphene-based devices with inkjet printing. The researchers successfully prepared graphene-contained inks, which were formed by liquid-based exfoliation of graphite powder with N-methyl-pyrrolidine. These functional inks were used to print thin-film transistors which had a mobility of approximately 95 cm^2^∙V∙s^−1^. The transmittance of these inks was approximately 80% of the transmittance of graphene and the resistance was approximately 30 kΩ∙cm^−2^. These inventions paved the way for the fabrication of printed, flexible, and transparent graphene-based electronics on a uniform substrate. Pristine GO ink was found to exhibit stability above 9 months at a 1 mg∙mL^−1^ concentration, with well-suited fluidic properties for effective and viable ink-jet printing devices [84]. A conductance of 9.24 × 10^3^ S∙m^−1^ was obtained after 30 printing stages at 300 °C. The resistance of the electrode printed on a flexible substrate was raised by < 5% after 1000 bending cycles and by 5.3% under a 180° bending angle. This reported technique for developing inks and conductive electrodes has proven to be promising for applications related to graphene-based flexible electronics. Homenick et al. [85] formulated a SWCNT-based ink via hybrid extraction-adsorption. The SWCNTs concentration, amount of ink incorporated, and comparative ratio of the polymer to SWCNTs were controlled using the SWCNTs’ network density. The optimized inkjet printing parameters were identified on Si/SiO_2_, where an ink with a polymer: SWCNTs ratio of 6:1 and 50 mg∙L^−1^ SWCNTs concentration printed at drops spaced 20 μm apart leads to a mobility of thin film transistor of approximately 25 cm^2^∙V∙s^−1^ with on-off voltage ratios greater than 10^5^. The mentioned conditions produced an efficient network regularity and was utilized in an additive process to synthesize TFT on a PET substrate, with motilities greater than 5 cm^2^∙V∙s^−1^. The ink-jet printing encapsulation layer effectively resulted in a TFT sample with a mobility of more than 1 cm^2^∙V∙s^−1^_;_ the use of inverter circuits resulted in stable and efficient operation conditions. Han et al. [86] reported the fabrication of a water-based conductive SWCNTs ink with sodium dodecyl-benzene sulfonate (surfactant). Direct writing on a paper with the prepared ink was performed with an off-the-shelf nib as well as a cartridge in the jet pen handwriting device. The lighter weight and moveable nature of the device meant that writing on a curved substrate was possible. The paper was obtained by a wetting approach. Double-sided and many-layered paper circuit boards were established via direct writing. The printed writing showed regularity and renewability. The extraordinary adhesion of SWCNTs on the cellulose paper showed efficient robustness against different mechanical loads or stress.

**Figure 7:**
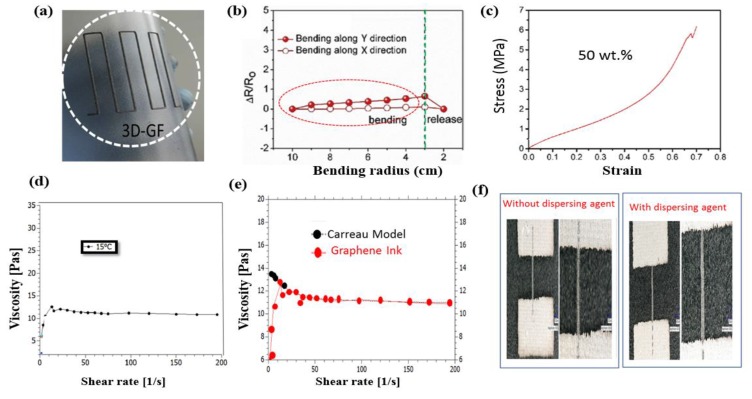
(**a**) Macrograph of 3D printed individual graphene filament, (**b**) electrical resistance of flexible circuit with 50 wt.% graphene content at various bending radius, and (**c**) typical stress strain curves of printed structure of 50 wt.% graphene loading, reproduced with permission from [88], published by Elsevier, 2018; (**d**) viscosity curves of graphene based ink with dispersing agent at 15 °C temperatures, (**e**) graphene ink with dispersing agent viscosity curves with fitted Carreau Model curve, and (**f**) graphene printed paths produced with or without dispersing agent graphene ink were printed via piezoelectric inkjet printer at following parameters, nozzle diameter (50 m), 40–50 V, pulse length (150–200 s), reproduced with permission from [89], published by MDPI, 2018.

Additionally, Foster et al. [87] reported on using graphene-based polylactic acid filaments to print 3D disc electrodes with a Rep-Rep FDM 3-D printer. The prepared 3D electrodes were characterized electrochemically and physiochemically. The prepared 3D electrode was applied as freestanding anodes in a Li^+^ battery and used in hydrogen generation via H-evolution reaction and exhibited tremendous catalytic activity. The results suggested that 3D printing of graphene-based conductive filaments enables the facile synthesis of energy storage devices. Recently, You et al. [88] demonstrated a direct ink-writing technique by preparing a 3D structure with stacked layers based on light-mass cellular interlinked networks. A homogenized graphene dispersion was formed via ultrasonication in ethanol. The speed of the printer and nozzle size were organized in such a way as to form 3D graphene; the material exhibited morphological stability, with 50% of the graphene contents in the filament, as mentioned in Figure 7a–c, and maintained the tremendous properties of graphene. This material was utilized as a 3D material in 3D printers. For the purpose of improving the printing properties, Sarapuk et al. [89] worked on the properties of graphene ink (including viscosity, strain, and shear ink rate) and presented the results via model-based calculations for an inkjet printer nozzle. The experimental study was conducted by preparing inks with or without a dispersing agent to validate the model. The results showed that the dispersing agent played a valuable role in improving the viscosity, printing power, and path with minimum resistivity, as shown in Figure 7d–f. Furthermore, stable graphene-based inks with a dispersing agent can produce effective prints.

### 3.3. Biosensors

**Figure 8:**
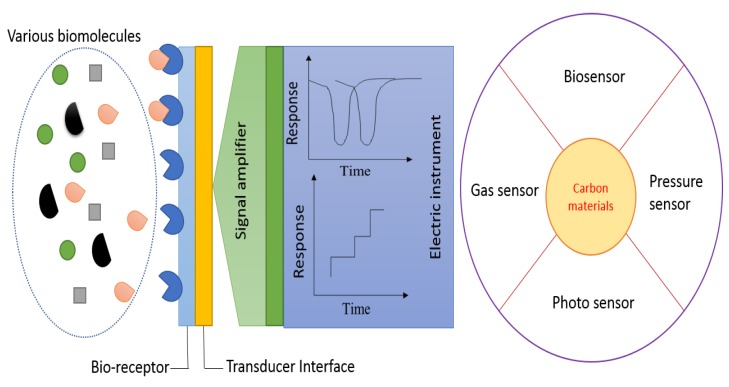
General layout of a typical biosensors system, and use of carbon materials in various sensors applications.

A biosensor is a machine that can detect molecules with a specific transducer to produce a detectable signal from the sample [140,141]. The schematic illustration is shown in Figure 8, indicating a general platform and the interface between a bio-receptor and a transducer.

The electrochemical-based biosensor contains 2–3 electrodes in a cell that are responsible for the transformation of a biological condition to electrochemical signals. It mostly contains biomolecules on an electrode that interact with analytes and lead to the generation of electrochemical signals [142]. Li et al. [90] fabricated a PDA-Lac-rGO-Pd composite for use as a sensing material. An electrochemical biosensor was prepared using composite to detect catechol. Under optimum conditions, the prepared electrochemical biosensor exhibited linearity in the range of 0.1–263 μM, with a sensitivity of 18.4 μAm∙M^−1^ at the minimum detection range of 0.03 μM. Furthermore, the electrochemical sensor possessed efficient repeatability, renewability, as well as stability. An identical electrochemical biosensor containing Lac exhibited the ability to detect minute amounts of catechol in unique water conditions. On the other hand, Hassan et al. [93] investigated a unique, effective glucose biosensor above functionalized rGO. This biosensor had a glucose-dependent electro-chemical behavior with Ag-AgCl as the standard electrode. This graphene-based biosensor with an enzyme possessed a very wide range of detection of glucose concentrations (up to 100 Mm), with sensitiveness of 5.59 μA∙D^−1^. These types of biosensors can be used as an efficient indicator of sugar level for biological diagnoses. Hemanth et al. [97] also reported an enzyme-focused electro-chemical biosensor. This biosensor was fabricated using a 3D pyrolytic carbon micro-electrode that was deposited with biologically functionalized rGO. The glucose-sensing properties of the 3D rGO-based biosensor were compared with those of 2D electrodes using cyclic voltammetry. The results showed that the 3D biosensor exhibited twice the sensing ability of the 2D electrodes i.e., with a value of 23.56 μAm∙(M∙cm^2^)^−1^ instead of 10.19 μAm∙(Mcm^2^)^−1^. The stability analysis of the enzyme above 3D rGO presented renewable results above 1 week. The prepared biosensor showed higher glucose selectivity over uric/ascorbic acid and cholesterol. 

Furthermore, CNT-based electrochemical biosensors also proved to be very efficient in sensing applications because of their tremendous merits, including great sensitiveness, rapid responsivity, ease of handling, and reliable transportability. A literature survey [143,144] indicated that CNT-based electrochemical biosensors have been investigated for the detection of biologically essential analytes by electro-chemical reaction catalyzed via different bio-enzymes [145], including glucose oxidases (GODs) [146], horse radish peroxidases [147], lactases [148], and malate dehydrogenases [149].

### 3.4. Gas Sensors

Gas sensors are very important components to sense the nature and amount of gas. A gas sensor can alter gas components and concentrations to obtain information based on the electric measurements of gas [150]. In 2014, a hybrid composite with rGO coated with ZnO nanomaterials was fabricated through a redox reaction [99]. The gas-sensing activities indicated that the response of the hybrid composites towards 5 ppm NO_2_ gas was 25.6% with a speed of 165 s and a reproducible time of 499 s. Furthermore, Liu et al. [151] reported on gas sensors with bimetallic nanoparticles (SnO_2_, ZnO) above graphene having a 3D porous morphology (pore size of 3–10 nm). This G/SnO_2_/ZnO composite gas sensor showed rapid and efficient NO_2_ gas adsorption response at various concentrations, with a response time of <1 min and efficient reproducibility (within 1 min). Similarly, Novikov et al. [100] successfully fabricated the cheapest ultrasensitive gas sensor based on an epitaxial graphene/silicon carbide composite. This sensor was functional under minimum operating concentrations (<1 ppb) with high sensitivity towards NO_2_ in an air mixture. A prototype of a replaceable electronic device to check surrounding levels of NO_2_ that uses a mixture of gases at ambient temperature was built. The prepared sensor can be recovered at room temperature and resulted in very rapid and reproducible analysis of NO_2_ (5–50 ppb). 

On the other hand, Huang and Hu [102] reported on the use of a graphene-doped polyaniline composite as a NH_3_ gas sensor. They found that the prepared hybrid sensor was capable of NH_3_ sensing with a response of 59.2% at 50 ppm concentration. The sensor also possessed an effective response towards H_2_, which was investigated by Zou et al. [152]. A t_res_ of 20 s and t_rec_ of 50 s were found when the prepared PANi-GO-based sensor was exposed to 1% (by volume) of H_2_ at ambient temperature. Recently, a hybrid composite was prepared with TiO_2_ (due to its high specific surface area and inexpensiveness) and graphene. This type of composite sensor proved to be very efficient to sense NH_3_ due to the existence of a large number of active adsorption sites [105]. A detailed literature survey on carbon-based composites and their functionalization for biosensors, gas sensors, and many other sensors are summarized in Table 2.

### 3.5. Wearable Electronic Devices 

**Figure 9:**
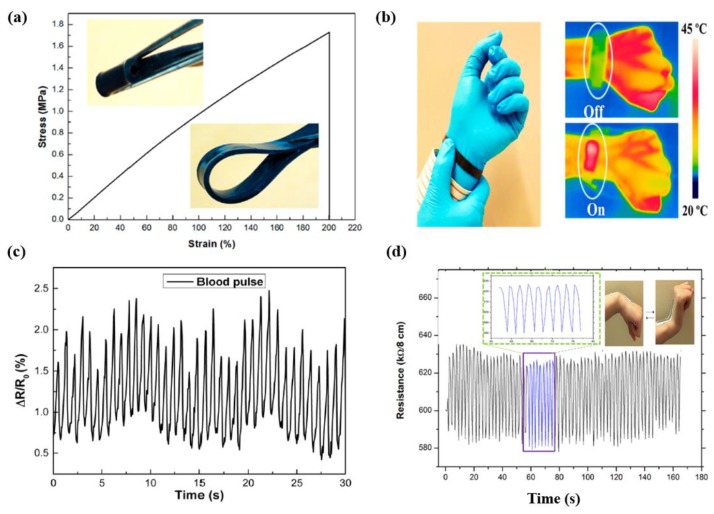
(**a**) Typical tensile strain-stress curve of CS/PDMS composite, (**b**) Infrared images of multifunctional wrist band worn by a volunteer as a wearable heater at on and off modes, (**c**) relative change of resistance (RCR) response of the wrist band to blood pulse of an adult volunteer; monitoring of the breathing, reproduced with permission from [110], published by ACS Publications, 2016; and (**d**) the upward/downward motion of wrist recorded by rGO-based cotton fabric sensors, and inset shows the magnified version of blue box in image (**d**) in the time range of 57–77 s, reproduced with permission from [111], published by ACS Publications, 2017.

There are broad varieties of flexible and stretchable materials available for utilization in different wearable appliances, including replaceable sensors and flexible electrodes and circuits. In the healthcare sector, wearable electronics have proven to be very efficient to check human health, movements, and thermo-therapy. Dinh et al. [170] designed CNT-based thermal sensors as wearable electronics for humans by utilizing lightweight high-strength stretchable CNT yarns as a heating wire, a graphitic pencil as an electrode, and minimum weight, durable, and bio-recyclable paper as a stretchable substrate. This CNT-based device was used as a sensor by fixing on human skin to monitor real-time respiration rates and to detect respiratory-related diseases. In addition, they also fixed a temperature detector within a similar sensor to analyze human body temperature in a contactless mode. This invention paved the way for the utilization of CNT yarns for the development of a broad range of eco-friendly, inexpensive, and lighter weight stretchable and wearable electronics related to temperature and breathing detectors. Zhan et al. [106] reported an identical, inexpensive SWCNT-tissue paper based stretchable wearable pressure sensor. This wearable pressure sensor showed tremendous performance and various advantages, such as greater sensitiveness for a wide range of pressures with minimum energy utilization (6–10 W), with real-time monitoring of various physical muscle activities. Additionally, the application of this pressure sensor to detect the response of force and pressure under synthetic robotic skin was also investigated. Wearable devices related to the detection of human movements and physical actions of athletes have also been developed. In this regard, Wang and Loh [107] fabricated multi-functional wearable sensors with a CNT-based fabric for monitoring the bending movements of human fingers in order to detect the commercial strain-sensing capability of the sensors. In addition, the CNT-fabric based sensor was fixed on a chest band to monitor the human breathing rate. This invented CNT-fabric-based sensor exhibited several merits, including flexibility, facile synthetic approach, low weight, inexpensiveness, and reliability for human use. Li et al. [110] fabricated a multi-functional wearable wristband prepared from CS/PDMS composites that exhibited high flexibility, confirmed by stress-strain curve as shown in Figure 9a. This wristband could act as a heater for thermotherapy, a biosensor for human blood pulse, and a breathing (shown in Figure 9c) and movements detector. The wristband could act as a heater below 15 V with a constant temperature variation of 20 °C. As a strain sensor, it exhibited rapid, reproducible response and efficient stability within a strain range of 0–20% and an employed frequency range of 0.01 to 10 Hz. The efficient flexibility, intermediate conductivity, efficient strain-sensing ability, and inexpensive nature make the multifunctional wearable CS/PDMS band a potential candidate for healthcare devices.

rGO-based e-textile wearables exhibit a wide range of benefits over conventional metal-based approaches. These conventional methods are complex and unsuitable for a wide range of practical applications. Therefore, Karim et al. [111] reported a facile, inexpensive technique to fabricate rGO-doped wearable devices through a facile pad-dry approach. This technique enabled the efficient manufacturing of conductive rGO e-textiles at an industrial fabrication rate of approximately 150 m∙min^−1^. The rGO-based e-textile electronic wearable devices possessed reliable softness, stability, and washability. The use of rGO increased the flexible nature and tensile strength of cotton fabrics by increasing the percentage of strain at the highest weight. The activity of the prepared rGO e-textile sensor was monitored by human wrist upward-downward motions and the results are shown in Figure 9d. 

This invented rGO e-textile wearable device was tested as a commercial sensor and heating appliance. The wearable tribo-electric sensor was compatible with an intelligent setup. However, the low conductivity, stability, and compatibility of e-textile electronics prohibited the fabrication of reliable incorporated generators for human clothing. To overcome these drawbacks, Wu et al. [112] fabricated wearable electronic generators based on polyester/Ag nanowires/graphene core-shell hybrids impregnated on an efficient, opaque, and smart e-textile via an eco-friendly full solution technique. The prepared smart e-textile device showed tremendous conductivity under a 20 Ω square and was efficient, flexible, stretchable, bendable, and washable. It can act as an electrode and wearable device, however due to its wearability, the smart e-textile generator was easily fitted in gloves to demonstrated the mechanical power produced by movements of fingers. The maximum recorded power by a single generator-based glove due to finger movement was 7 nW∙cm^−2^. The properties of this composite-based smart e-textile prove that it as an efficient candidate for practical wearable clothing.

## 4. Conclusions 

In this review, we have summarized a large number of research papers related to the fabrication of nanocomposites materials based on chemically functionalized GO/rGO/GS, SWCNTs, MWCNTs, AC, and CFs. The outstanding properties of these functionalized carbon allotrope-based composites, including high specific surface area, high durability, high thermal and electrical conductivity, high resistivity, and elastic flexibility, have resulted in remarkable applications in the electronic industry. MO-doped, hybrids, and composites of carbon materials have improved the electronic properties of devices. This review also elaborated the recent development of carbon allotrope composites and their applications in energy storage devices, supercapacitors, ink formulation, inkjet-printing devices, bio-gas and pressure sensors, as well as stretchable/flexible wearable electronics. Overall, the results indicated that the properties of carbon allotropes can be improved and polished by fabricating composites and hybrids, which broaden their applications in the commercial electronic industry. 

## 5. Future Outlooks

As mentioned in the literature survey, different hybrid materials, methodologies, and techniques have been applied to produce energy storage electronics that exhibit high specific capacitance, energy density, and power density. It is well known that carbon-based hybrids are an efficient contender for electrodes in supercapacitors; however, they require supportive dynamic materials to increase their efficiency. The surface modification of electrodes by carbon allotropes (CNTs, AC, CFs, and rGO) functionalized by conducting materials, such as polymers, MO, and magnetic nanoparticles, significantly enhances the power and energy density, specific capacitance, and reusability of supercapacitor electrodes. If further research efforts are devoted to this field, then functionalized carbon allotrope-based supercapacitors can be expected to be a novel innovation in the field of electronics.

On the other hand, important developments have been made in inkjet 3D printing by using graphene- and SWCNT-based inks. Many newly designed inkjet 3D printers exhibit performance superior to that of earlier-generation printers. Conventional approaches have not been frequently adopted by a large number of industries because of practical issues related to efficiency, price, and reliability of bulk material formation. For solvent-based direct-writing techniques in inkjet printers, the rheology of inks is essential. In order to manufacture reliable inks, adequate filler contents with a homogeneous dispersion are mandatory. Furthermore, a small concentration of graphene-based inks has been utilized to prepare 3D graphene-based tools, but has recently been deemed inappropriate for broad-range production because of its harsh print settings. Therefore, the proper selection of printing technique and materials are still existing issues. The use of carbon-based inks still requires optimized conditions in order to improve the homogeneity, stabilization, and fine-printing ability at an industrial level. 

Furthermore, electrodes functionalized with SWCNTs, MWCNTs, or different graphene forms with changeable band-gaps seem to be more reliable for the fabrication of biosensors. These sensors are able to sense different biological components, including protein, glucose, and enzymes, with great sensitivity because of their high flexibility and specific surface area. These carbon-based sensors also have the ability of detecting gases (O_2_, N_2_, NH_3_, and H_2_). However, a facile approach for controlled fabrication and easy handling of graphene should be an important research topic in the upcoming years. Recent chemical methodologies to functionalize graphene with biological compounds have proven to be efficient in enhancing the detecting efficiency of the targeted components, although the refining of the detecting surface material is required in order to restrict the adsorption of non-relevant species during the sensing performance. In addition, miniaturized compact-type biosensor devices having high durability, accuracy, sensitivity, and cost efficiency are highly demanded for the detection of viruses and bacteria to monitor the human health situation.

A large number of applications of functionalized carbon-based stretchable devices for the monitoring of human health and body movements have been developed. This includes the observation of nerve pulse, respiratory rate, and body heat. The development of multifunctional wearable electronics remains limited and should be given greater consideration. In the near future, the simultaneous use of functionalized carbon-based composites and biocompatible components for efficient wearable devices may be possible. Smart electronic devices have also attracted attention recently. In the near future, more progress in advanced carbon-based stretchable devices will highly contribute to the development of more efficient devices for the health-care and medical improvement sectors.

## Figures and Tables

**Table 1:** Different carbon forms, their modification and application in supercapcitors. GNS: graphene nanosheet, KTP: Korean traditional paper, pErGO: porous electrochemically reduced graphene oxide, Cuf; copper foil, AC; activated carbon.

Carbon Forms	Functionalization	Specific Capacitance (F/g)	Electrolyte (M)	Retention Cycle	References
MWCNTs	PANI (20%)	670	H_2_SO_4_ (1)	-	[119]
MWCNTs	Ppy	427	Na_2_SO_4_ (1)	-	[120]
CNTs	M-PANI	1030	H_2_SO_4_ (1)	5000	[121]
MCNTs	PEDOT	120	H_2_SO_4_ (1)	20000	[122]
Graphene	PANI(80%)	320	H_2_SO_4_ (2)	-	[123]
Graphene	PANI	1126	H_2_SO_4_ (1)	1000	[124]
GNS	PANI	1130	H_2_SO_4_ (1)	1000	[125]
pErGO	Cuf/Cu wire	81	PVA/H_3_PO_4_	5000	[126]
AC	Fe_3_O_4_	37.9	KOH (6)	500	[127]
CNTs	RuO_2_-TiO_2_	50	KOH (1)	1000	[128]
Carbon black	Fe_3_O_4_	5.3	Na_2_SO_4_ (1)	10000	[129]
3D GO	PANI	1341	H_2_SO_4_ (1)	5000	[130]
N-doped-rGO	PANI	610	H_2_SO_4_ (1)	1000	[131]
rGO	PANI-Co_3_O_4_	1063	KOH (6)	2500	[132]
rGO	PANI, ZrO_2_	1360	H_2_SO_4_ (1)	1000	[133]
B-doped rGO	PANI	406	H_2_SO_4_ (1)	10000	[134]
MWCNT	Ni_3_S_2_	55.8	KOH (2)	5000	[135]
graphene	MoS_2_	268	Na_2_SO_4_ (1)	1000	[136]
CNTs	CuS	112	KOH (2)	1000	[137]

**Table 2:** Carbon-based hybrids, their functionalization/modification as sensors.

Carbon Material	Modification	Analyte	Detection Limit	References
MWCNTs	COOH	O_2_	0.3%	[153]
MWCNTs	maleic acid, acetylene	NH_3_	10 ppm	[154]
SWCNTs	Pd doping/sputtering	H_2_	0.5%	[155]
SWCNTs	LaFeO_3_	methanol	1 ppm	[156]
SWCNTs	Pd nanoparticles	glucose	0.2 mM	[157]
MWCNTs	Pt nanoparticles	glucose	1 × 10^−5^ mol/L	[158]
Multi-layered graphene	Poly(vinylpyrrolidone), glucose oxidase	glucose	2 mM	[159]
rGO	Sulfophenyl, ethylenediamine	NO_2_	3.6 ppm	[160]
rGO	Au-Pt alloy, chitosan-glucose oxidase	glucose	5 mM	[161]
Graphene foam	α-Fe_2_O_3_	NO_2_	0.12 mM	[162]
GO	poly(3,4-ethylenedioxythiophene)	dopamine	0.33 mM	[163]
rGO	PNF-AgNPs	H_2_O_2_	10.4 μM.	[164]
GO	peptide-AgNPs	H_2_O_2_	0.13 mM	[165]
Graphene foam	CuO nanoflower	ascorbic acid	0.43 mM	[166]
rGO	CeO_2_/GCE	NO_2_	9.6 nM	[167]
rGO	AuFe_3_O_4_/Pt	H_2_O_2_	0.1 nM	[168]
GO	Au@Pt@Au NPs	H_2_O_2_	0.02 nM	[169]
